# Protein tyrosine phosphatase, PTP1B, expression and activity in rat corneal endothelial cells

**Published:** 2007-05-24

**Authors:** Deshea L. Harris, Nancy C. Joyce

**Affiliations:** Schepens Eye Research Institute and Department of Ophthalmology, Harvard Medical School, Boston, MA

## Abstract

**Purpose:**

The current studies were conducted to determine whether the protein tyrosine phosphatase, PTP1B, plays a role in regulating epidermal growth factor receptor (EGFR) Tyr992 phosphorylation and cell cycle entry in rat corneal endothelial cells.

**Methods:**

Corneas were obtained from male Sprague-Dawley rats. PTP1B mRNA and protein expression were compared in confluent and subconfluent cells by RT-PCR and western blots. Immunocytochemistry was used to determine the subcellular localization of both PTP1B and EGFR following epidermal growth factor (EGF) stimulation. Western blots were used to analyze the time-dependent effect of EGF on phosphorylation of EGFR Tyr992 plus or minus CinnGEL 2Me, an inhibitor of PTP1B activity. The effect of PTP1B inhibition on cell cycle entry was determined by calculating the percent of Ki67-positive cells following EGF treatment.

**Results:**

PTP1B mRNA expression was similar in confluent and subconfluent cells, but PTP1B protein was expressed at 3 fold higher levels in subconfluent cells. Positive staining for PTP1B was localized in vesicular structures below the plasma membrane. EGFR staining was located at cell-cell borders in untreated endothelium, but was mainly cytoplasmic by 15 min after EGF treatment. In control cultures, phosphorylation of EGFR Tyr992 peaked by 5 min following EGF stimulation and rapidly decreased to basal levels by 30 min. In cultures pretreated with CinnGEL 2Me, Tyr992 phosphorylation peaked 2 min following EGF addition and was consistently sustained at a higher level than controls until 60 min after treatment. By 18 h following EGF treatment, cultures pretreated with CinnGEL 2Me exhibited a 1.7 fold increase in the number of Ki67-positive cells compared with control cultures.

**Conclusions:**

Comparison of PTP1B mRNA and protein levels indicates that PTP1B expression is regulated mainly at the protein level and is higher in subconfluent cells. PTP1B was located in vesicles below the plasma membrane. The fact that EGFR is internalized in response to EGF stimulation suggests that it could interact with and be regulated by PTP1B. The ability of PTP1B inhibitor to sustain EGFR Tyr992 phosphorylation and increase the number of Ki67-positive cells indicates that PTP1B plays a role in the negative regulation of EGF-induced signaling and helps suppress cell cycle entry.

## Introduction

Corneal endothelial cells do not normally proliferate in vivo to increase cell numbers. However, they retain proliferative capacity and can divide both in culture and in ex vivo corneas if cell-cell contacts are disrupted and cells are exposed to positive growth factors [1,2]. Epidermal growth factor (EGF) has been shown to induce proliferation in corneal endothelial cells from several species, including rabbits [3], cows [4,5], cats [6,7], non-human primates [8,9], and humans [8,10-12]. Although EGF is known to stimulate proliferation in these cells, there is very little information regarding how the EGF-induced signal is regulated.

The EGF receptor (EGFR) is an 1,186 amino acid transmembrane protein and is a member of a group of receptors possessing intrinsic tyrosine kinase activity [13]. Reversible tyrosine phosphorylation helps regulate important cellular processes, including proliferation, migration, and differentiation [14]. In response to ligand binding, specific tyrosine residues within the COOH-terminal intracellular domain of EGFR become autophosphorylated. These residues include Tyr992 and Tyr1148 [15]. Tyrosine autophosphorylation within growth factor receptors promotes direct binding of signaling proteins that contain src homology-2 (SH2) domains [15-18]. Ligand binding to EGFR can lead to activation of a number of signaling pathways, including phospholipase C-γ (PLC-γ) and its downstream calcium- and protein kinase C (PKC) cascades, and ras which leads to activation of various MAP kinases. Upon ligand binding and activation, EGFR is rapidly internalized into endosomes, with its extracellular domain within the endosome and its intracellular domain extending toward the cytoplasm. EGFR remains active in the endosome for several min before either being sorted to lysosomes (where it is degraded) or recycled back to the plasma membrane [19]. The fate of the receptor and the output of the signaling process depend on continued ligand binding and kinase activity [13,20].

The catalytic activity of many receptor tyrosine kinases is tightly regulated by protein tyrosine phosphatases (PTPs), which act as "on" and "off" switches for numerous signaling events [14,21]. PTP1B is a widely expressed 50 kDa non-receptor PTP [22] that helps regulate multiple cellular functions, including proliferation. Among its functions, PTP1B binds to the EGFR both in vitro [15] and in vivo [23] and specifically interacts with and dephosphorylates both Tyr992 and Tyr1148 within the cytoplasmic domain of the receptor [15]. Studies indicate that there is competition for PTP1B binding at these sites. For example, the SH2 domain-containing protein, PLC-γ, also interacts with Tyr992, while the GTPase-activating protein of ras (GAP) interacts with Tyr1148. This competitive binding indicates that PTP1B must play a role in regulating EGFR downstream signaling via PLC-γ and/or GAP. PTP1B contains a 35 amino acid COOH-terminal hydrophobic sequence that anchors it to the cytoplasmic surface of the endoplasmic reticulum [24]. PTP1B-catalyzed dephosphorylation of EGFR and attenuation of the phosphotyrosine-induced downstream signal requires receptor endocytosis and occurs in a specific "dephosphorylation compartment" prior to the movement of the receptor to lysosomes or to recycling back to the plasma membrane [14,25,26].

A number of studies have been conducted to determine whether manipulating the expression or activity of PTP1B can have an effect on growth factor-induced downstream cellular processes. For example, PTP1B attenuated PDGF-BB-induced proliferation in cultured vascular smooth muscle cells via dephosphorylation of the PDGF-beta receptor, whereas expression of a dominant-negative form of PTP1B promoted proliferation of these cells [27]. In immortalized fibroblasts from PTP1B^-/-^ mice, cells lacking PTP1B exhibited increased and sustained phosphorylation of both EGFR and the PDGF receptor [28]. PTP1B also negatively regulates phosphotyrosine-induced signaling via the insulin and leptin receptors, implicating PTP1B activity in Type 2 diabetes and obesity [29-31]. Because of the potential role of PTP1B in these diseases, as well as in cancer, much attention has been paid to the development of specific inhibitors of PTP1B as potential therapeutic agents [14,32,33].

Studies from this laboratory [34] have demonstrated the expression of several PTPs in rat corneal endothelial cells both in ex vivo corneas and in culture. Among the PTPs expressed were PTP1B, SHP-1, SHP-2, PTP-mu, and the dual-specificity phosphatase, PTEN. When cultures of rat corneal endothelial cells were treated with the general phosphatase inhibitor, sodium orthovanadate (SOV), cell cycle entry was increased, as indicated by an increase in the number of cells with positive staining for Ki67, a marker of actively cycling cells [35]. The increase in Ki67-positive cells upon exposure to SOV strongly suggests that phosphatase activity helps suppress cell cycle entry in corneal endothelium. The current studies were conducted to determine whether PTP1B plays a role in regulating Tyr992 phosphorylation of EGFR and cell cycle entry in rat corneal endothelial cells.

## Methods

### Rat corneal tissue

Corneas were obtained from adult male Sprague-Dawley rats (Taconic, Hudson, NY), which were treated in accordance with the ARVO Statement on the Use of Animals in Ophthalmic and Vision Research. Whole corneas were used for some experiments. For other experiments, endothelial cell explant cultures were prepared according to Chen et al. [36]. Primary cultures were grown to confluence in Medium 199 (Invitrogen, Carlsbad, CA) supplemented with 50 μg/ml gentamicin (Invitrogen) and 10% fetal bovine serum (FBS; HyClone, Logan, UT). All incubations were performed at 37 °C in a 5% CO_2_, humidified atmosphere. Passage 2 cells were used for all experiments.

### RT-PCR of PTP1B

Total RNA was prepared from passage 2 subconfluent and confluent cultures of rat corneal endothelial cells according to the manufacturer's directions (TRIzol; Invitrogen). cDNA was prepared from 1 μg total RNA by reverse transcription in a volume of 20 μl using reagents from a commercially available kit (Promega, Pittsburgh, PA). Primers specific for PTP1B were available from Santa Cruz Biotechnology (Santa Cruz, CA). Primer sequences were based on mouse PTP1B sequences. PCR was performed according to protocols provided by Santa Cruz and included two PCR reactions. Briefly, the first PCR reaction was performed in a mixture containing 100 ng cDNA and 1 μl of Primer Pair A, plus reagents from a commercially available kit (Qiagen, Valencia, CA). Specificity and yield of the PCR products were enhanced using the hot-start approach [37]. PCR was performed for 36 cycles. Cycle conditions included denaturation at 94 °C for 30 s, annealing at 55 °C for 30 s, and extension at 72 °C for 3 min. A 10 min extension was added at the end of the 36 cycles of PCR. A second PCR reaction was performed in a mixture containing 1 μl of product from the first reaction and 1 μl of Primer Pair B, plus reagents from the aforementioned commercially available kit. After the hot start, PCR was performed for 26 cycles, and the conditions were the same as above except that the extension time at 72 °C was for only 1.5 min. Negative controls consisted of samples containing all reagents plus primers, but without cDNA. Glyceraldehyde-3-phosphate dehydrogenase (G3PDH; Clontech, Mountain View, CA) acted as a positive control for the PCR. To ensure that the total RNA samples were not contaminated with genomic DNA, a negative control using 100 ng total RNA was substituted for cDNA in the PCR reaction mixture, along with 2.5 μM each of upstream and downstream G3PDH primers. PCR products and 100 bp DNA ladder molecular weight markers were electrophoresed in 1.5% agarose gels containing 0.5 μg/ml ethidium bromide and photographed. All experiments were conducted in duplicate and repeated three times to ensure consistent results.

### Protein extraction and western blot analysis of PTP1B

Passage 2 subconfluent and confluent cultures of rat corneal endothelial cells were used for western blot analysis of PTP1B protein levels. Protein was extracted by incubating cells for 30 min at 4 °C in lysis buffer containing 1% Triton X-100, 250 mM NaCl, 2 mM EDTA, 50 mM Tris-HCl (pH 7.4), 10 μg/ml aprotinin, 10 μg/ml leupeptin, 1 mM phenylmethylsulfonyl fluoride, 50 mM sodium fluoride, and 0.1 mM sodium orthovanadate (all from Sigma, St. Louis, MO), followed by sonication and centrifugation. Supernatants were stored at -80 °C until used for SDS-PAGE and western blotting. Equal concentrations of soluble protein were loaded on 10% Bis-Tris gels (Invitrogen) for SDS-PAGE and then transferred to a polyvinylidene difluoride (PVDF) membrane (Millipore, Bedford, MA). Nonspecific binding was blocked by incubating the membrane overnight at 4 °C in 5% non-fat dry milk diluted in phosphate-buffered saline (PBS; Invitrogen). The membrane was incubated for 2 h at room temperature in PTP1B mouse monoclonal antibody (PH01: Calbiochem/EMD Biosciences, San Diego, CA) at a concentration of 1.2 mg/ml diluted in 5% non-fat dry milk in PBS. Blots were then rinsed 2 times for 10 min each with PBS containing 0.1% Triton X-100 and incubated 1 h at room temperature with HRP-conjugated donkey anti-mouse IgG (Jackson ImmunoResearch Laboratories, West Grove, PA) diluted at 1:10,000 in 5% non-fat dry milk in PBS. Membranes were washed 2 times for 10 min with PBS containing 0.1% Triton X-100. Antibody binding was visualized using a chemiluminescent substrate (SuperSignal West Pico; Pierce, Rockford, IL). To prepare a control for protein loading, bound antibodies were stripped from the PVDF membrane by incubation in buffer containing 2% SDS, 62.5 mM Tris-HCl (pH 6.8) and 100 mM 2-mercaptoethanol (all from Sigma) for 15 min at room temperature. The membrane was reprobed with β-actin mouse monoclonal antibody (Sigma) diluted at 1:10,000 in 5% non-fat dry milk in PBS followed by incubation in HRP-conjugated donkey anti-mouse IgG diluted at 1:20,000 in 5% non-fat dry milk in PBS. A positive control sample was prepared from SW480 cells, a human colorectal adenocarcinoma cell line (American Type Culture Collection, Manassas, VA). Semi-quantitative analysis of protein expression was performed by densitometry using NIH Image software (NIH Image 1.34; National Institutes of Health, Bethesda, MD). Expression of PTP1B was normalized relative to that of β-actin. Duplicate immunoblots were prepared and each experiment was repeated three times.

### Immunocytochemical localization of PTP1B

Rat corneas were washed in PBS and then fixed with 100% methanol for 10 min at -20 °C. All subsequent steps were performed at room temperature. Corneas were washed 3 times in PBS for 10 min each, then permeabilized for 10 min with 1% Triton X-100 in PBS, and washed again 3 times in PBS for 10 min each. Nonspecific binding was blocked using 4% bovine serum albumin (BSA; Fisher, Pittsburgh, PA) in PBS for 10 min. Corneas were incubated for 2 h in PTP1B mouse monoclonal antibody diluted 1:50 in 4% BSA in PBS and at the same time in β-catenin goat polyclonal antibody (Santa Cruz Biotechnology) diluted 1:100 in 4% BSA in PBS. Corneas were washed 3 times in PBS for 10 min each and then incubated for 1 h with rhodamine red-X donkey anti-mouse IgG diluted 1:100 in 4% BSA in PBS for detection of PTP1B antibody and FITC donkey anti-goat IgG diluted at 1:50 to detect β-catenin antibody. Both secondary antibodies were obtained from Jackson ImmunoResearch Laboratories. Negative controls consisted of secondary antibody alone. After being washed in PBS 3 times for 10 min each, corneas were placed endothelial-side up on slides using mounting medium (Vector Laboratories, Burlingame, CA). Digital images were obtained using a Leica TSC-SP2 confocal microscope (Bannockburn, IL). A Z-series through the tissue was captured with a step size of 0.5 μm per image. Z-series images were collapsed onto a single image plane by projecting the maximal pixel intensity of the images.

### Immunocytochemical localization of EGFR following EGF stimulation

Rat corneas were washed and incubated for 0, 15, and 60 min in Medium 199, gentamicin, 0.1% FBS plus 10 ng/ml epidermal growth factor (EGF; Upstate Biotechnology, Charlottesville, VA). The corneas were then washed in PBS and fixed with 100% methanol for 10 min at -20 °C. All subsequent steps were performed at room temperature. Corneas were washed 3 times in PBS for 10 min each, then permeabilized for 10 min with 1% Triton X-100 in PBS, and washed again 3 times in PBS for 10 min each. Nonspecific binding was blocked using 4% BSA in PBS for 10 min. Corneas were incubated for 2 h in EGF receptor (EGFR) sheep polyclonal antibody diluted 1:30 in 4% BSA in PBS or ZO-1 mouse monoclonal antibody (Zymed/Invitrogen) diluted 1:150 in 4% BSA in PBS. Corneas were washed 3 times in PBS for 10 min each and then incubated for 1 h with FITC donkey anti-sheep IgG diluted at 1:50 for detection of EGFR or rhodamine red-X donkey anti-mouse IgG diluted 1:200 in 4% BSA in PBS for detection of ZO-1. Both secondary antibodies were obtained from Jackson ImmunoResearch Laboratories. Negative controls consisted of secondary antibody alone. After being washed in PBS 3 times for 10 min each, corneas were placed endothelial-side up on slides using mounting medium (Vector Laboratories). Digital images were obtained as described above.

### Western blot analysis of effect of PTP1B inhibitor on EGFR Tyr992 levels

Confluent passage 1 rat corneal endothelial cells were trypsinized, seeded in 60 mm petri dishes at a density required to yield 50% confluence, and incubated overnight in the presence of 10% serum to permit cell attachment. Cells were then serum-starved for 24 h in Medium 199 plus gentamicin. For initial testing of the EGFR Tyr992 time-course, cells were incubated with EGF at a concentration of 25 ng/ml in Medium 199 and gentamicin without serum for 0, 10, 20, 30, 40, 50, or 60 min. These control experiments were repeated 3 times. CinnGEL 2Me, a novel peptoid inhibitor of PTP1B [38], was used to test the effect of PTP1B inhibition on the time-course of EGFR Tyr992 phosphorylation. CinnGEL 2Me was reconstituted in dimethyl sulphoxide (DMSO: Sigma) as indicated by the manufacturer (BioMol Research Laboratories, Plymouth Meeting, PA). Cells were incubated in the presence of CinnGEL 2Me at a final concentration of 1 mM or in an equal volume of DMSO in Medium 199 and gentamicin without serum for 1 h prior to the start of the experiment. EGF was then added at a concentration of 25 ng/ml to the CinnGEL 2Me or DMSO-containing medium and cells were incubated for 0, 2, 5, 15, 30, 60, or 120 min. At each time-point, dishes were placed on ice and cells were washed with ice-cold PBS. Protein extraction, gel electrophoresis, and protein transfer to PVDF membranes were performed as described above. Nonspecific binding was blocked by incubation of the membrane for 1 h at room temperature in 5% non-fat dry milk and 0.1% Triton X-100 diluted in PBS. The membrane was briefly washed with 0.1% Triton X-100 in PBS and then incubated overnight at 4 °C with EGFR Tyr992 rabbit polyclonal antibody (Cell Signaling Technology, Danvers, MA) diluted at 1:500 in 5% BSA and 0.1% Triton X-100 in PBS. Blots were then rinsed 2 times for 10 min each with PBS containing 0.1% Triton X-100 and incubated 1 h at room temperature with HRP-conjugated donkey anti-rabbit IgG (Jackson ImmunoResearch Laboratories) at a 1:2,000 dilution in 5% non-fat dry milk and 0.1% Triton X-100 diluted in PBS. Membranes were washed 2 times with PBS containing 0.1% Triton X-100 for 10 min and antibody binding was visualized using a chemiluminescent substrate (SuperSignal West Femto; Pierce). To prepare a control for protein loading, bound antibodies were stripped as described above. The membrane was re-probed with β-actin mouse monoclonal antibody (Sigma) diluted at 1:10,000 in 5% non-fat dry milk in PBS followed by incubation in HRP-conjugated donkey anti-mouse IgG diluted in 5% non-fat dry milk in PBS at 1:20,000. Quantification of protein bands was performed by densitometry as described above. β-Actin was used for normalization. Experiments comparing CinnGEL 2Me and DMSO controls were repeated 4 times. Averaged data is presented ± standard error of the mean (SEM).

### Analysis of PTP1B inhibitor on cell cycle entry

Confluent passage 1 rat corneal endothelial cells were trypsinized as above and seeded into 4-well glass chamber slides (Lab-Tek II, Nagle Nunc International, Rochester, NY) coated with FNC Coating Mix (AthenaES, Baltimore, MD) to yield 50% confluence. Cells were incubated in Medium 199, gentamicin, and 0.1% FBS overnight and then the medium was changed to 0% FBS for 24 h. CinnGEL 2Me (1 mM final concentration) or DMSO was added to the medium for 1 h prior to the start of the experiment. EGF was added to all wells at a final concentration of 25 ng/ml and cells were incubated for 0, 6, or 18 h. At each time point, the slides were processed for immunocytochemistry as follows: Cells were washed quickly 3 times with PBS and then fixed with 100% methanol for 10 min at -20 °C. All subsequent steps were performed at room temperature. Cells were washed 3 times in PBS for 10 min each and then permeabilized for 10 min with 1% Triton X-100 in PBS. Cells were washed 3 times in PBS for 10 min each. Nonspecific binding was blocked using 4% BSA in PBS. Cells were incubated for 2 h in Ki67 mouse monoclonal antibody (Novocastra, Newcastle upon Tyne, UK) diluted at 1:50 in 4% BSA in PBS. Cells were washed 3 times in PBS for 10 min each and then incubated for 1 h in FITC-conjugated donkey anti-mouse IgG diluted at 1:50 in 4% BSA in PBS. The cells were washed in PBS 3 times for 10 min each and prepared in mounting medium containing propidium iodide (PI: Vector Laboratories). Digital fluorescent images were obtained using an Eclipse E800 Nikon Microscope with VFM Epi-Fluorescence Attachment (Nikon, Inc., Melville, NY) equipped with a Spot digital camera and Spot Advanced version 4.5 CE software (Diagnostic Instruments, Sterling Heights, MI). Ki67 and PI-positive cells were counted from 3 different areas in duplicate wells per experiment and the average percent of Ki67-positive cells was calculated. Averaged data is presented ±SEM.

## Results

### Expression of PTP1B in confluent and subconfluent cells

**Figure 1:**
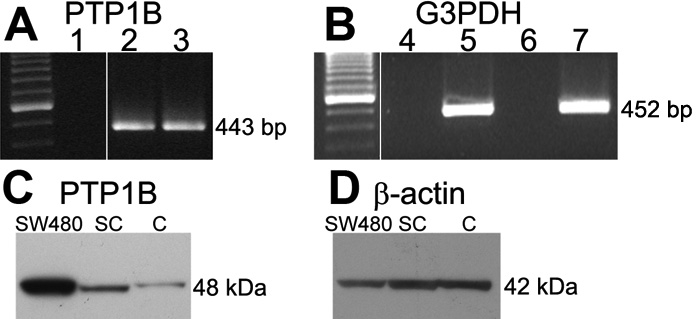
Representative results comparing PTP1B mRNA and protein expression in confluent and subconfluent rat corneal endothelial cells. **A**: RT-PCR for PTP1B. At left are molecular weight markers in 100 bp increments with the brightest band at 600 bp. Lane 1: Control sample containing all reagents, but without cDNA; Lane 2: cDNA extracted from confluent cells; Lane 3: cDNA extracted from subconfluent cells. Position of 443 bp band for PTP1B is indicated. **B**: RT-PCR for G3PDH control. At left are molecular weight markers; Lane 4: Control sample containing total RNA extracted from confluent cells; Lane 5: cDNA extracted from confluent cells; Lane 6: Control sample containing total RNA extracted from subconfluent cells; Lane 7: cDNA extracted from subconfluent cells. Position of 452 bp G3PDH band is indicated. **C**: Western blot for PTP1B (48 kDa) showing position of PTP1B in control SW480 cells and relative expression of PTP1B in subconfluent (SC) and confluent (C) rat corneal endothelial cells. **D**: Same membrane as in (**C**) reprobed for β-actin to control for protein load.

Previous studies [34] provided immunocytochemical and western blot evidence that rat corneal endothelial cells express PTP1B. The current studies confirmed and extended this finding by comparing relative mRNA and protein expression for PTP1B in confluent versus subconfluent rat corneal endothelial cells. Cells were maintained in the presence of 10% FBS prior to processing for RT-PCR and western blots. RT-PCR revealed positive bands for PTP1B and G3PDH for both confluent and subconfluent cells (Figure 1A,B). Controls containing all reagents plus PTP1B primers, but without cDNA, were negative. Samples containing total RNA rather than cDNA yielded no positive bands, indicating lack of contamination of the RNA samples with genomic DNA. Band density for PTP1B was very similar in both confluent and subconfluent cells. In this study, PCR was performed using two sets of primer pairs based on methods recommended by the supplier. To confirm these results, PCR of the second set of primers was run for 10, 20, and 26 cycles for both samples. Similar results were obtained under all cycle conditions (data not shown) and indicate that PTP1B mRNA is expressed at similar levels in both confluent and subconfluent cells. Western blots (Figure 1C,D) showed a strong positive band for PTP1B in the positive control, as well as positive bands for both corneal endothelial samples. In contrast to the RT-PCR data, densitometric analysis of the western blots indicates that PTP1B is expressed at 3 fold higher levels in subconfluent versus confluent cells. Experiments were performed 3 times and similar results were obtained for both the RT-PCR and western blots in each experiment.

### PTP1B localization in endothelial cells of ex vivo rat corneas

**Figure 2:**
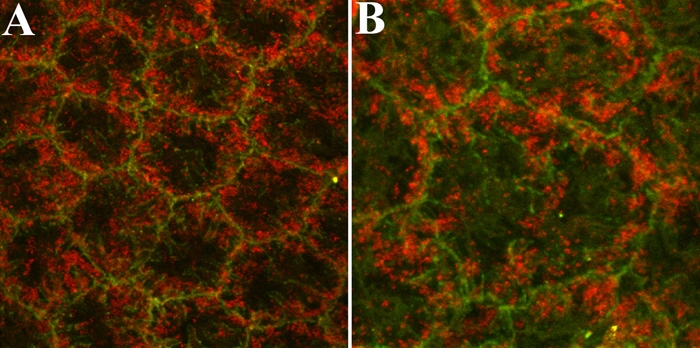
Representative fluorescent confocal images showing immunostaining for PTP1B and β-catenin in corneal endothelial cells of ex vivo rat corneas. PTP1B (red) localization is compared with that of β-catenin (green), a protein closely associated with cell-cell adhesion junctions. The staining pattern of PTP1B strongly suggests that PTP1B is associated with vesicular structures subjacent to the plasma membrane. The higher magnification image in (**B**) is included to more clearly demonstrate this localization pattern. The final magnification in **A** was 2100X and in **B** was 3120X.

Although previous studies [34] provided evidence that PTP1B is expressed in rat corneal endothelial cells, they did not clearly determine its subcellular location. Confocal images in Figure 2 show positive staining for PTP1B in a punctate pattern suggestive of cytoplasmic vesicles. Comparison of the PTP1B staining pattern with staining for β-catenin, a protein associated with cell-cell adhesion junctions [39], indicates that PTP1B is associated with vesicular structures immediately subjacent to the plasma membrane. Tissue incubated in secondary antibody alone was negative in all cases (data not shown).

### Localization of EGFR in rat corneal endothelial cells in response to EGF stimulation

**Figure 3:**
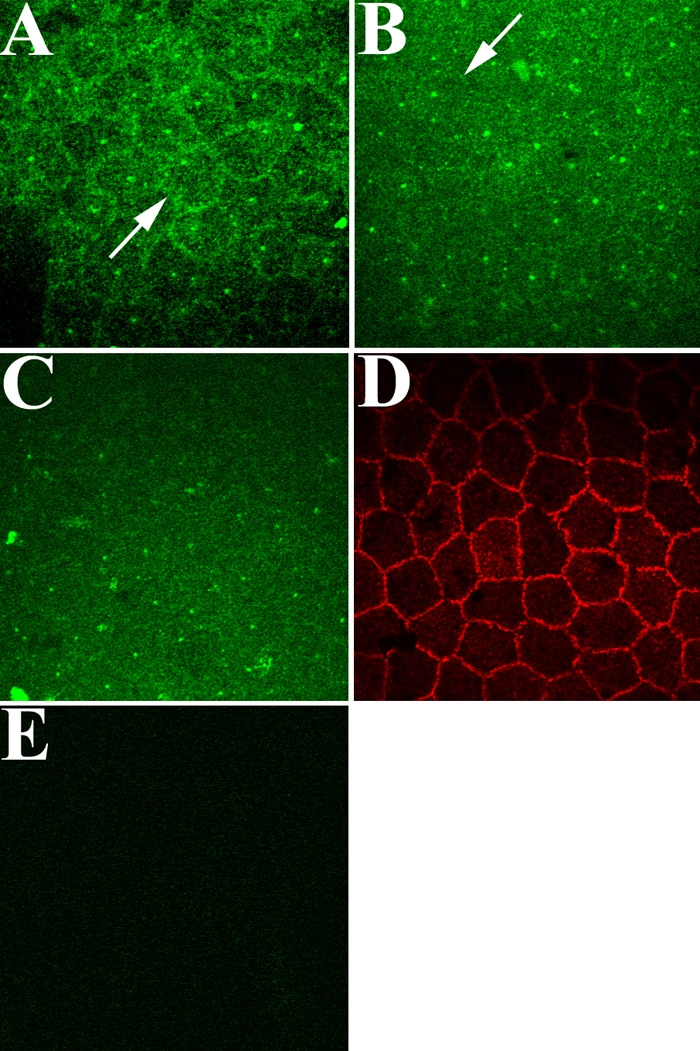
Fluorescent confocal images of EGFR localization in corneal endothelial cells of ex vivo rat corneas following EGF stimulation. In the absence of EGF, EGFR is located mainly at cell borders (arrow), although some cytoplasmic punctate staining is visible (**A**). After EGF stimulation for 15 min (**B**), EGFR staining is greatly reduced at cell borders (arrow). After 60 min (**C**), little-to-no EGFR is visible at cell borders; however, ZO-1 staining of the same tissue (**D**) indicates that cell borders remain intact. No staining is observed in the endothelium of ex vivo corneas incubated in secondary antibody alone (**E**). Note that small dots of intense stain can be observed scattered in the cytoplasm of cells in (**A**-**C**). The specific nature of this staining is unclear, since all antibodies were centrifuged at high speed prior to dilution to prevent nonspecific antibody deposition. Final magnification: 1000X.

Immunocytochemical localization studies were conducted to determine the effect of EGF treatment on the localization of EGFR. Ex vivo rat corneas were incubated in the presence of EGF for 0, 15, and 60 min. Corneas were fixed at each time point, immunostained for EGFR, and analyzed by confocal microscopy. Representative images are shown in Figure 3. In the absence of EGF (Figure 3A), endothelial cells exhibited intense staining for EGFR at cell borders and diffuse punctate staining throughout the cell interior. Within 15 min after exposure to EGF (Figure 3B), there was a visible reduction in EGFR staining at lateral membranes (although cell borders could still be seen in some areas) and the diffuse punctate cytoplasmic staining appeared to increase. By 60 min after EGF addition (Figure 3C), little-to-no staining for EGFR was visible at cell borders; however, staining for ZO-1, a tight junction-associated protein [39] in the same tissue (Figure 3D) indicated that cell-cell contacts remained intact in the presence of EGF.

### Time-course of EGFR Tyr992 phosphorylation

**Figure 4:**
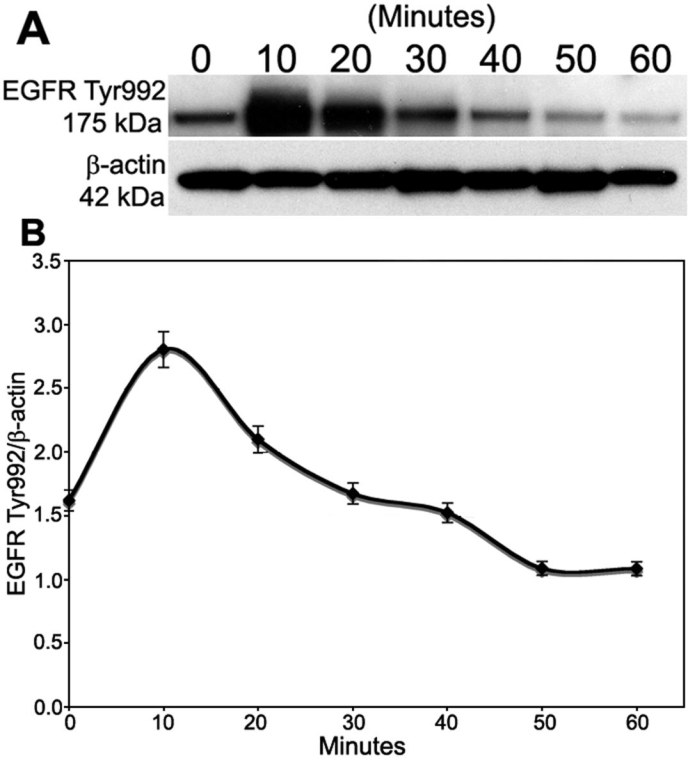
Time-course of EGFR Tyr992 phosphorylation. Subconfluent rat corneal endothelial cells were serum-starved and then treated with 25 ng/ml EGF in Medium 199 plus gentamicin. Samples were taken at various times for western blot analysis. **A**: Representative western blot showing the time-course of EGFR Tyr992 phosphorylation. Phosphorylated EGFR Tyr992 yields a 175 kDa band. β-Actin (42 kDa) was used for a loading control. **B**: Graph showing the average level of phosphorylated EGFR Tyr992 in duplicate gels from three separate experiments. β-Actin was used for normalization. Bars represent SEM.

Studies were first conducted to determine the effect of EGF on the time-course of phosphorylation of EGFR Tyr992. Serum-starved subconfluent rat corneal endothelial cells were treated with 25 ng/ml EGF in Medium 199 plus gentamicin. This concentration of EGF was chosen based on preliminary dose-response studies (data not shown). Samples were then taken for western blot analysis of phosphorylated Tyr992 at 0, 10, 20, 30, 40, 50, or 60 min following EGF addition and results are shown in Figure 4. Figure 4A presents a representative western blot showing the time-course of EGFR Tyr992 phosphorylation following EGF stimulation. Densitometric analysis of the blots (Figure 4B) indicates that phosphorylated Tyr992 levels peaked by 10 min following EGF addition and decreased to basal levels by 30 min.

### Effect of PTP1B inhibitor on time-course of EGFR Tyr992 phosphorylation

**Figure 5:**
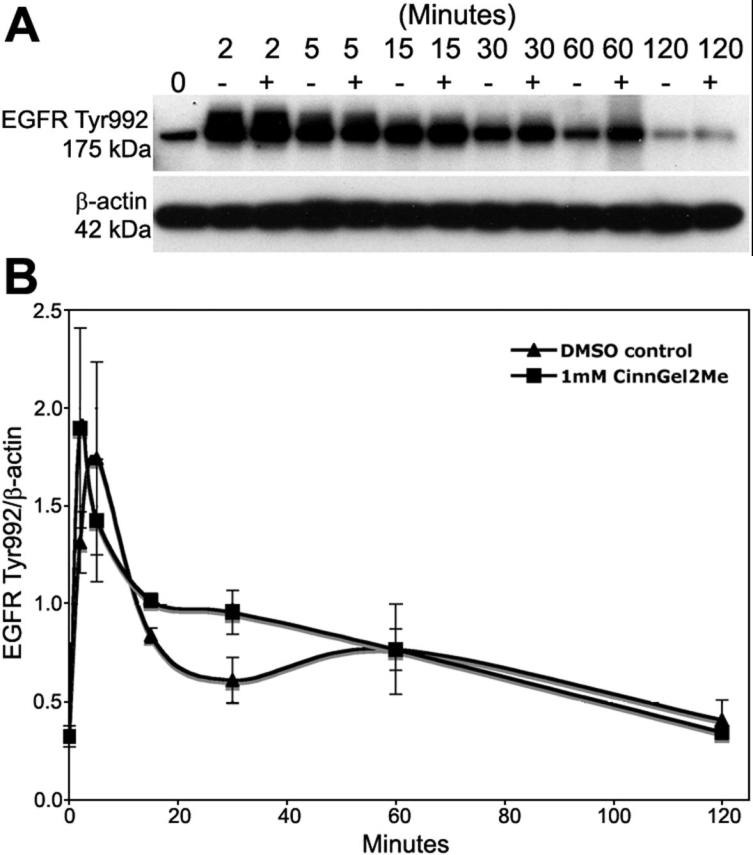
Effect of the PTP1B inhibitor, CinnGEL 2Me, on the time-course of EGFR Tyr992 phosphorylation. Serum-starved, subconfluent rat corneal endothelial cells were pre-incubated for 1 h in Medium 199, gentamicin, and DMSO(-) or supplemented with 1 mM CinnGel 2Me(+). EGF (25 ng/ml) was then added and samples were taken for western blot analysis at various times following EGF addition. Representative western blot in (**A**) shows phosphorylated Tyr992 at 175 kDa. Densitometric analysis was conducted using β-actin for normalization. The graph in (**B**) shows average levels of phosphorylated Tyr992. Results are the average of duplicate gels from four separate experiments. Bars represent SEM.

Studies were then conducted to determine the effect of CinnGEL 2Me, a peptoid inhibitor of PTP1B, on the time-course of Tyr992 phosphorylation. CinnGEL 2Me is the methyl ester of a tripeptide-substituted cinnamic acid that exhibits significant inhibition of PTP1B activity [38]. The supplier of CinnGEL 2Me states that the methyl ester permits cell permeability and is hydrolyzed to active inhibitor by intracellular esterases. For this study, subconfluent cultures of rat corneal endothelial cells were serum-starved for 24 h and then preincubated for 1 h in culture medium containing Medium 199, gentamicin, and DMSO either with or without 1 mM CinnGEL 2Me. DMSO was used as a control for these experiments, because the inhibitor was solubilized in this reagent. The concentration of CinnGEL 2Me was chosen following a preliminary dose-response study (data not shown). After this 1 h incubation, cultures were treated with 25 ng/ml EGF for 0, 2, 5, 15, 30, 60, or 120 min and samples were taken for western blot analysis. Figure 5A presents representative results of the western blots and Figure 5B shows the results of the densitometric analysis. Overall, the kinetics of EGFR Tyr992 phosphorylation in cells maintained in control medium containing DMSO were very similar to those described above in which cells were maintained in medium without DMSO. This indicates that DMSO did not affect the ability of the cells to respond to EGF stimulation. In the DMSO-treated control cells, phosphorylated Tyr992 levels peaked 5 min after EGF addition, rapidly decreased to near-basal levels by 30 min, and then gradually returned to basal levels by 120 min. In CinnGEL 2Me-treated cells, Tyr992 phosphorylation peaked 2 min after EGF addition. Although the relative level of phosphorylation decreased over time, it was consistently sustained at a level higher than that observed in controls until the 60 min time-point. Thus, inhibition of PTP1B by CinnGEL 2Me resulted in a more rapid response to EGF stimulation and an increased duration of EGFR Tyr992 phosphorylation.

### Effect of PTP1B inhibitor on cell cycle entry

**Figure 6:**
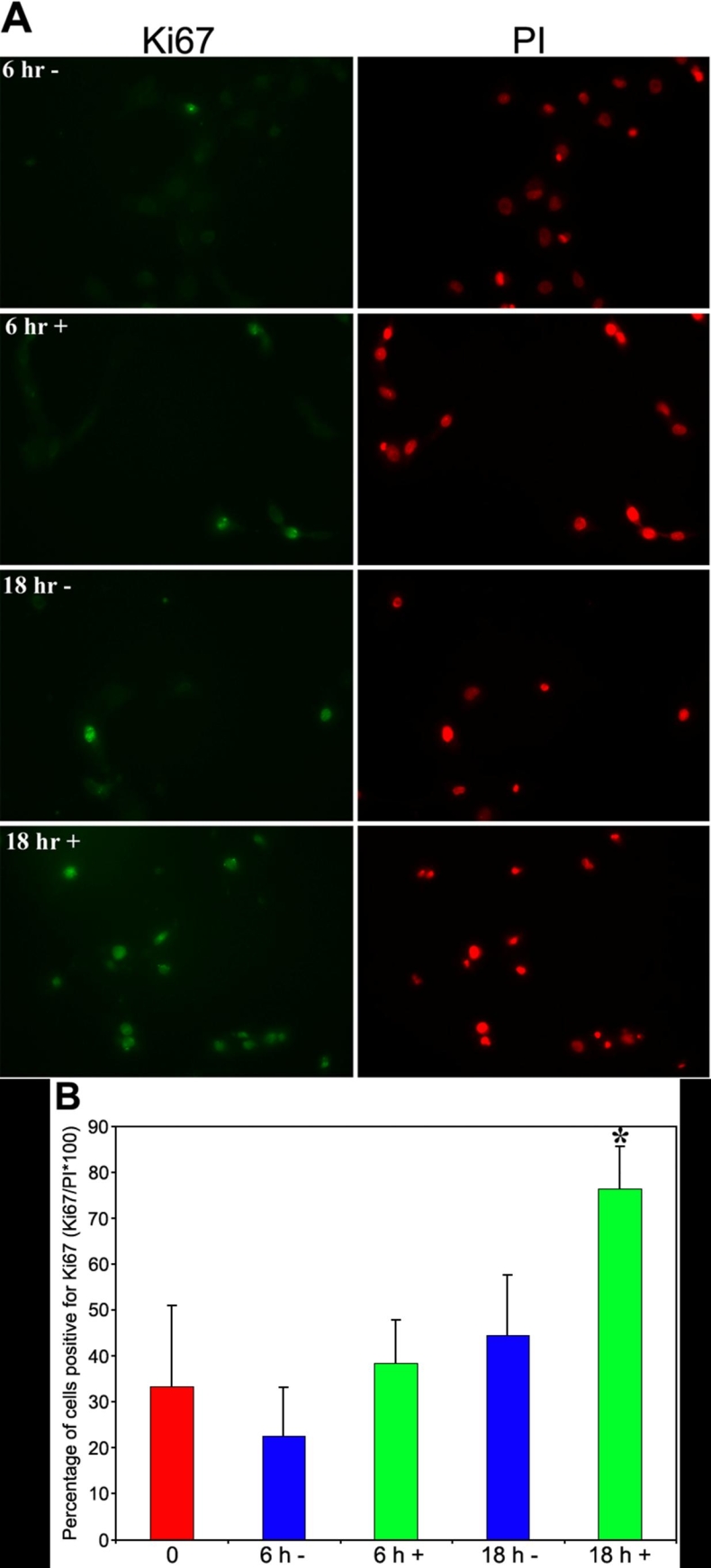
Effect of CinnGEL 2Me inhibition of PTP1B on cell cycle entry. Serum-starved subconfluent rat corneal endothelial cells were preincubated for 1 h in medium containing either DMSO(-) or 1 mM CinnGel 2Me in DMSO(+) followed by addition of 25 ng/ml EGF for 6 or 18 h. **A**: Cultures were immunostained for Ki67 (green), a marker of proliferating cells, and counterstained with propidium iodide (PI) to reveal all nuclei (red). Magnification: 400X. **B**: Bar graph shows the average number of Ki67-positive cells at each time-point in duplicate samples from three separate experiments. Bars represent SEM and the asterisk indicates a 1.7 fold increase over control.

Studies were also conducted to determine the effect of PTP1B inhibitor on EGF-stimulated cell cycle entry. For these studies, subconfluent rat corneal endothelial cells were serum-starved, pre-treated with or without 1 mM CinnGEL 2Me, and then stimulated with 25 ng/ml EGF as previously. Cells were then immunostained for Ki67, a marker of actively cycling cells [35], at 0, 6, and 18 h after EGF addition. Cells positively stained for Ki67 and total propidium iodide (PI)-stained nuclei were counted and the results compared. Figure 6A presents representative fluorescent images of Ki67- and PI-stained cells at 6 and 18 h after EGF treatment, while Figure 6B shows the percent of Ki67-positive cells averaged from duplicate samples in three separate experiments. As expected from the normal time-course of cell cycle progression in rat corneal endothelial cells [36], there were no Ki67-positive cells at the 0 h time-point (data not shown) and there was a low number of Ki67-positive cells at the 6 h time-point under both treatment conditions. By 18 h after EGF treatment, the average number of Ki67-positive cells was increased at least 1.7 fold in cultures treated with CinnGEL 2Me compared with cultures incubated in control medium.

## Discussion

This laboratory is the first to explore a role for protein tyrosine phosphatases in the regulation of growth factor signaling in corneal endothelium from any species. In a previous study [34] we used rat corneal endothelium to identify a subset of PTPs expressed in these cells and to examine the effect of SOV-induced PTP inhibition on cell cycle entry. PTP1B was among the PTPs identified in that study. PTP1B is widely expressed, but its specific function(s) within individual cell types is still being investigated [14]. PTP1B is clearly involved in the regulation of signal transduction and has been found to play an important role in diabetes, obesity, the cell cycle, and cancer [40]. Since PTP1B is known to regulate EGFR signaling and corneal endothelial cells express these receptors, it is important to obtain a better understanding of the role of this PTP in EGF-induced cell cycle traverse. The current studies were conducted in rat corneal endothelial cells, because the initial studies were conducted in this species and it was important to obtain a base of information in rat that could eventually be applied to studies of PTP1B in human corneal endothelial cells. The goal of these studies was to determine whether PTP1B plays a role in regulating EGF-induced Tyr992 phosphorylation of EGFR and cell cycle entry in rat corneal endothelial cells.

The current studies confirmed and extended the finding that PTP1B is expressed in rat corneal endothelial cells. PTP1B protein levels were found to be substantially higher in subconfluent cells compared with confluent cells, while PTP1B mRNA levels were similar, strongly suggesting that PTP1B expression is regulated mainly at the protein level in these cells. This finding is interesting, because subconfluent cells were used in studies testing the effect of the PTP1B inhibitor. Protein tyrosine phosphorylation is normally balanced by dephosphorylation, suggesting that the relative expression of PTPs may be dependent on overall tyrosine phosphorylation activity. In fact, several studies have demonstrated that expression of PTP1B is increased upon neoplastic transformation [41,42], as well as in cancer [43,44]. Overall, our finding of increased PTP1B protein levels in subconfluent cells appears consistent with increased proliferative activity. The specific mechanism by which PTP1B protein levels are regulated in corneal endothelial cells remains to be determined. PTP1B has also been shown to regulate both cadherin- [45] and integrin-based adhesion in some cells [46,47]. The relative difference in PTP1B protein levels in confluent versus subconfluent cells may also reflect these functions of PTP1B in corneal endothelial cells, but this remains to be established.

The PTP1B staining pattern in the endothelium suggests it is associated with submembranous vesicles; however, the current studies did not test whether PTP1B is specifically associated with the endoplasmic reticulum as has been reported in other cell types [24]. The fact that EGFR is internalized in response to EGF stimulation suggests that it could interact with and be regulated by PTP1B. Indeed, the similarity in the kinetics of EGFR internalization and the kinetics of Tyr992 phosphorylation supports this idea. The kinetics of EGFR Tyr992 phosphorylation in cells incubated in culture medium containing DMSO were very similar to those of cells incubated in medium without DMSO, indicating that DMSO did not significantly affect EGF-induced signaling, at least under these experimental conditions. The finding that inhibition of PTP1B activity by CinnGEL 2Me results in sustained EGFR Tyr992 phosphorylation compared with controls is similar to that of Haj, et al. [28], who found that fibroblasts isolated from PTP1B^-/-^ mice exhibited increased and sustained phosphorylation of EGFR, as well as of the receptor for PDGF. Together, results strongly suggest that PTP1B negatively regulates EGF-induced receptor signaling in rat corneal endothelial cells.

Previous studies found that treatment of rat corneal endothelial cells with the general phosphatase inhibitor, SOV, increased the number of Ki67-positive cells over controls, indicating that phosphatase inhibition promoted cell cycle entry. These results were similar to those reported by Suzuki, et al. [48], who showed that SOV treatment promoted cell cycle entry in cultured vascular endothelial cells. The current study extends this finding by demonstrating that treatment of subconfluent rat corneal endothelial cells with CinnGEL 2Me increased the number of Ki67-positive cells, thereby implicating PTP1B in the negative regulation of EGF-induced cell cycle entry. The current studies did not explore the effect of CinnGEL 2Me on the extent of cell cycle traverse. Positive staining for Ki67 is first upregulated in mid-G_1_-phase of the cell cycle [49,50]. As such, the observed increase in the number of Ki67-positive cells in CinnGEL 2Me treated cultures suggests that more cells had at least begun to enter the cell cycle. In the previous study of rat corneal endothelial cells treated with SOV, it appeared that inhibition of phosphatases was not sufficient to move cells from mid-G_1_ through S-phase of the cycle, since there was no increase found in the number of BrdU-stained cells. It is possible that inhibition of PTP1B is sufficient to promote changes leading to the initial stages of cell cycle entry, but that other signaling mechanisms compensate for the lack of PTP1B activity, resulting in the inability of cells to further traverse the cycle. This possibility was suggested by Haj et al. [28] who observed an increase in EGFR phosphorylation in PTP1B^-/-^ mice, but detected little-to-no increase in the activity of downstream signals, suggesting the existence of compensatory mechanisms to prevent uncontrolled growth factor receptor activation. Further study will be needed to more specifically identify the changes induced by PTP1B inhibition that promote cell cycle entry. In addition, careful studies are needed to identify the specific downstream signaling cascades affected by PTP1B activity. It is assumed that PLC-γ and the GAP-MAP kinase cascades will be affected, since these proteins appear to compete for PTP1B binding for phosphotyrosines on EGFR in other cell types. It should also be mentioned that EGF is not the only growth factor known to stimulate proliferation in corneal endothelial cells. For example, significant information has been obtained in rabbit corneal endothelial cells regarding cell cycle regulation by fibroblast growth factor-2 (FGF-2) [51,52]. It would be interesting to determine whether PTP1B might help regulate FGF-2 signaling in these cells, as has been found in cultured smooth muscle cells [27].

In summary, results strongly suggest that PTP1B plays a role in the negative regulation of EGFR signaling in rat corneal endothelial cells, at least at the level of Tyr992 phosphorylation, and that inhibition of PTP1B activity increases both the duration of EGFR autophosphorylation and the relative number of cells entering the cell cycle. With this base of information, studies can now be conducted to explore the role of PTP1B in regulation of EGFR signaling in human corneal endothelium.

## References

[1] Joyce NC Proliferative capacity of the corneal endothelium. Prog Retin Eye Res 2003 22 359 89 12852491 10.1016/s1350-9462(02)00065-4

[2] Joyce NC Cell cycle status in human corneal endothelium. Exp Eye Res 2005 81 629 38 16054624 10.1016/j.exer.2005.06.012

[3] Jumblatt MM Matkin ED Neufeld AH Pharmacological regulation of morphology and mitosis in cultured rabbit corneal endothelium. Invest Ophthalmol Vis Sci 1988 29 586 93 2451652

[4] Savion N Isaacs JD Shuman MA Gospodarowicz D Proliferation and differentiation of bovine corneal endothelial cells in culture. Metab Pediatr Syst Ophthalmol 1982 6 305 20 6764247

[5] Woost PG Jumblatt MM Eiferman RA Schultz GS Growth factors and corneal endothelial cells: II. Characterization of epidermal growth factor receptor from bovine corneal endothelial cells. Cornea 1992 11 11 9 1559342 10.1097/00003226-199201000-00002

[6] Soltau JB McLaughlin BJ Effects of growth factors on wound healing in serum-deprived kitten corneal endothelial cell cultures. Cornea 1993 12 208 15 8500333 10.1097/00003226-199305000-00005

[7] Raphael B Kerr NC Shimizu RW Lass JH Crouthamel KC Glaser SR Stern GA McLaughlin BJ Musch DC Duzman E Conway J Schultz GS Enhanced healing of cat corneal endothelial wounds by epidermal growth factor. Invest Ophthalmol Vis Sci 1993 34 2305 12 8505212

[8] Fabricant R Salisbury JD Berkowitz RA Kaufman HE Regenerative effects of epidermal growth factor after penetrating keratoplasty in primates. Arch Ophthalmol 1982 100 994 5 7046709 10.1001/archopht.1982.01030031002022

[9] Nayak SK Samples JR °JK, Binder PS. Growth characteristics of primate (baboon) corneal endothelium in vitro. Invest Ophthalmol Vis Sci 1986 27 607 11 3485613

[10] Samples JR Binder PS Nayak SK Propagation of human corneal endothelium in vitro effect of growth factors. Exp Eye Res 1991 52 121 8 2013296 10.1016/0014-4835(91)90252-a

[11] Blake DA Yu H Young DL Caldwell DR Matrix stimulates the proliferation of human corneal endothelial cells in culture. Invest Ophthalmol Vis Sci 1997 38 1119 29 9152231

[12] Zhu C Joyce NC Proliferative response of corneal endothelial cells from young and older donors. Invest Ophthalmol Vis Sci 2004 45 1743 51 15161835 10.1167/iovs.03-0814

[13] Wells A EGF receptor. Int J Biochem Cell Biol 1999 31 637 43 10404636 10.1016/s1357-2725(99)00015-1

[14] Tonks NK PTP1B: from the sidelines to the front lines! FEBS Lett 2003 546 140 8 12829250 10.1016/s0014-5793(03)00603-3

[15] Milarski KL Zhu G Pearl CG McNamara DJ Dobrusin EM MacLean D Thieme-Sefler A Zhang ZY Sawyer T Decker SJ Dixon JE Saltiel AR Sequence specificity in recognition of the epidermal growth factor receptor by protein tyrosine phosphatase 1B. J Biol Chem 1993 268 23634 9 7693694

[16] Alroy I Yarden Y The ErbB signaling network in embryogenesis and oncogenesis: signal diversification through combinatorial ligand-receptor interactions. FEBS Lett 1997 410 83 6 9247128 10.1016/s0014-5793(97)00412-2

[17] Raab G Klagsbrun M Heparin-binding EGF-like growth factor. Biochim Biophys Acta 1997 1333 F179 99 9426203 10.1016/s0304-419x(97)00024-3

[18] Marshall CJ Specificity of receptor tyrosine kinase signaling: transient versus sustained extracellular signal-regulated kinase activation. Cell 1995 80 179 85 7834738 10.1016/0092-8674(95)90401-8

[19] Baass PC Di Guglielmo GM Authier F Posner BI Bergeron JJ Compartmentalized signal transduction by receptor tyrosine kinases. Trends Cell Biol 1995 5 465 70 14732031 10.1016/s0962-8924(00)89116-3

[20] Miaczynska M Pelkmans L Zerial M Not just a sink: endosomes in control of signal transduction. Curr Opin Cell Biol 2004 16 400 6 15261672 10.1016/j.ceb.2004.06.005

[21] Zhang ZY Zhou B Xie L Modulation of protein kinase signaling by protein phosphatases and inhibitors. Pharmacol Ther 2002 93 307 17 12191622 10.1016/s0163-7258(02)00199-7

[22] Chernoff J Schievella AR Jost CA Erikson RL Neel BG Cloning of a cDNA for a major human protein-tyrosine-phosphatase. Proc Natl Acad Sci USA 1990 87 2735 9 2157211 10.1073/pnas.87.7.2735 PMC53765

[23] Liu F Chernoff J Protein tyrosine phosphatase 1B interacts with and is tyrosine phosphorylated by the epidermal growth factor receptor. Biochem J 1997 327 139 45 9355745 10.1042/bj3270139 PMC1218773

[24] Frangioni JV Beahm PH Shifrin V Jost CA Neel BG The nontransmembrane tyrosine phosphatase PTP-1B localizes to the endoplasmic reticulum via its 35 amino acid C-terminal sequence. Cell 1992 68 545 60 1739967 10.1016/0092-8674(92)90190-n

[25] Haj FG Verveer PJ Squire A Neel BG Bastiaens PI Imaging sites of receptor dephosphorylation by PTP1B on the surface of the endoplasmic reticulum. Science 2002 295 1708 11 11872838 10.1126/science.1067566

[26] Yudushkin IA Schleifenbaum A Kinkhabwala A Neel BG Schultz C Bastiaens PI Live-cell imaging of enzyme-substrate interaction reveals spatial regulation of PTP1B. Science 2007 315 115 9 17204654 10.1126/science.1134966

[27] Chang Y Ceacareanu B Zhuang D Zhang C Pu Q Ceacareanu AC Hassid A Counter-regulatory function of protein tyrosine phosphatase 1B in platelet-derived growth factor- or fibroblast growth factor-induced motility and proliferation of cultured smooth muscle cells and in neointima formation. Arterioscler Thromb Vasc Biol 2006 26 501 7 16373608 10.1161/01.ATV.0000201070.71787.b8

[28] Haj FG Markova B Klaman LD Bohmer FD Neel BG Regulation of receptor tyrosine kinase signaling by protein tyrosine phosphatase-1B. J Biol Chem 2003 278 739 44 12424235 10.1074/jbc.M210194200

[29] Goldstein BJ Ahmad F Ding W Li PM Zhang WR Regulation of the insulin signalling pathway by cellular protein-tyrosine phosphatases. Mol Cell Biochem 1998 182 91 9 9609118

[30] Zabolotny JM Bence-Hanulec KK Stricker-Krongrad A Haj F Wang Y Minokoshi Y Kim YB Elmquist JK Tartaglia LA Kahn BB Neel BG PTP1B regulates leptin signal transduction in vivo. Dev Cell 2002 2 489 95 11970898 10.1016/s1534-5807(02)00148-x

[31] Cheng A Uetani N Simoncic PD Chaubey VP Lee-Loy A McGlade CJ Kennedy BP Tremblay ML Attenuation of leptin action and regulation of obesity by protein tyrosine phosphatase 1B. Dev Cell 2002 2 497 503 11970899 10.1016/s1534-5807(02)00149-1

[32] Guo XL Shen K Wang F Lawrence DS Zhang ZY Probing the molecular basis for potent and selective protein-tyrosine phosphatase 1B inhibition. J Biol Chem 2002 277 41014 22 12193602 10.1074/jbc.M207347200

[33] Zhang ZY Lee SY PTP1B inhibitors as potential therapeutics in the treatment of type 2 diabetes and obesity. Expert Opin Investig Drugs 2003 12 223 33 12556216 10.1517/13543784.12.2.223

[34] Chen WL Harris DL Joyce NC Effects of SOV-induced phosphatase inhibition and expression of protein tyrosine phosphatases in rat corneal endothelial cells. Exp Eye Res 2005 81 570 80 15950220 10.1016/j.exer.2005.03.015

[35] Gerdes J Li L Schlueter C Duchrow M Wohlenberg C Gerlach C Stahmer I Kloth S Brandt E Flad HD Immunobiochemical and molecular biologic characterization of the cell proliferation-associated nuclear antigen that is defined by monoclonal antibody Ki-67. Am J Pathol 1991 138 867 73 2012175 PMC1886092

[36] Chen KH Harris DL Joyce NC TGF-beta2 in aqueous humor suppresses S-phase entry in cultured corneal endothelial cells. Invest Ophthalmol Vis Sci 1999 40 2513 9 10509644

[37] Erlich HA Gelfand D Sninsky JJ Recent advances in the polymerase chain reaction. Science 1991 252 1643 51 2047872 10.1126/science.2047872

[38] Moran EJ Sarshar S Cargill JF Shahbaz MM Lio A Radiofrequency tag encoded combinatorial library method for the discovery of tripeptide-substituted cinnamic acid inhibitors of the protein tyrosine phosphatase PTP1B. J Am Chem Soc 1995 117 10787 8

[39] Petroll WM Hsu JK Bean J Cavanagh HD Jester JV The spatial organization of apical junctional complex-associated proteins in feline and human corneal endothelium. Curr Eye Res 1999 18 10 9 10075198 10.1076/ceyr.18.1.10.5392

[40] Dube N Tremblay ML Involvement of the small protein tyrosine phosphatases TC-PTP and PTP1B in signal transduction and diseases: from diabetes, obesity to cell cycle, and cancer. Biochim Biophys Acta 2005 1754 108 17 16198645 10.1016/j.bbapap.2005.07.030

[41] Zhai YF Beittenmiller H Wang B Gould MN Oakley C Esselman WJ Welsch CW Increased expression of specific protein tyrosine phosphatases in human breast epithelial cells neoplastically transformed by the neu oncogene. Cancer Res 1993 53 2272 8 8097963

[42] LaMontagne KR Jr Hannon G Tonks NK Protein tyrosine phosphatase PTP1B suppresses p210 bcr-abl-induced transformation of rat-1 fibroblasts and promotes differentiation of K562 cells. Proc Natl Acad Sci USA 1998 95 14094 9 9826659 10.1073/pnas.95.24.14094 PMC24332

[43] Wiener JR Kerns BJ Harvey EL Conaway MR Iglehart JD Berchuck A Bast RC Jr Overexpression of the protein tyrosine phosphatase PTP1B in human breast cancer: association with p185c-erbB-2 protein expression. J Natl Cancer Inst 1994 86 372 8 7905928 10.1093/jnci/86.5.372

[44] Bjorge JD Pang A Fujita DJ Identification of protein-tyrosine phosphatase 1B as the major tyrosine phosphatase activity capable of dephosphorylating and activating c-Src in several human breast cancer cell lines. J Biol Chem 2000 275 41439 46 11007774 10.1074/jbc.M004852200

[45] Balsamo J Arregui C Leung T Lilien J The nonreceptor protein tyrosine phosphatase PTP1B binds to the cytoplasmic domain of N-cadherin and regulates the cadherin-actin linkage. J Cell Biol 1998 143 523 32 9786960 10.1083/jcb.143.2.523 PMC2132848

[46] Arregui CO Balsamo J Lilien J Impaired integrin-mediated adhesion and signaling in fibroblasts expressing a dominant-negative mutant PTP1B. J Cell Biol 1998 143 861 73 Erratum in: J Cell Biol 1998; 143:1761 9813103 10.1083/jcb.143.3.861 PMC2148148

[47] Liang F Lee SY Liang J Lawrence DS Zhang ZY The role of protein-tyrosine phosphatase 1B in integrin signaling. J Biol Chem 2005 280 24857 63 15866871 10.1074/jbc.M502780200

[48] Suzuki E Nagata D Yoshizumi M Kakoki M Goto A Omata M Hirata Y Reentry into the cell cycle of contact-inhibited vascular endothelial cells by a phosphatase inhibitor. Possible involvement of extracellular signal-regulated kinase and phosphatidylinositol 3-kinase. J Biol Chem 2000 275 3637 44 10652360 10.1074/jbc.275.5.3637

[49] Kill IR Localisation of the Ki-67 antigen within the nucleolus. Evidence for a fibrillarin-deficient region of the dense fibrillar component. J Cell Sci 1996 109 1253 63 8799815 10.1242/jcs.109.6.1253

[50] Starborg M Gell K Brundell E Hoog C The murine Ki-67 cell proliferation antigen accumulates in the nucleolar and heterochromatic regions of interphase cells and at the periphery of the mitotic chromosomes in a process essential for cell cycle progression. J Cell Sci 1996 109 143 53 8834799 10.1242/jcs.109.1.143

[51] Kay EP Gu X Smith RE Corneal endothelial modulation: bFGF as direct mediator and corneal endothelium modulation factor as inducer. Invest Ophthalmol Vis Sci 1994 35 2427 35 8163333

[52] Gu X Seong GJ Lee YG Kay EP Fibroblast growth factor 2 uses distinct signaling pathways for cell proliferation and cell shape changes in corneal endothelial cells. Invest Ophthalmol Vis Sci 1996 37 2326 34 8843917

